# Influence of ACEs on Sleep Health in Early Childhood as Mediated by Family Routines

**DOI:** 10.3390/children13070949

**Published:** 2026-07-20

**Authors:** Ann H. Johnson, Sara L. Davis, David Phillipi, Mahdiyeh Ahmadi, Alexandra Armstrong, Elizabeth Coleman, Laura Gray, Shameka Rodgers Phillips, Heather C. Soistmann, Marti Rice

**Affiliations:** 1 Harris College of Nursing and Health Sciences, Texas Christian University, 2800 W Bowie St., Fort Worth, TX 76109, USA; 2 College of Nursing, University of South Alabama, Mobile, AL 36688, USA; saradavis@southalabama.edu; 3 Gordon E. Inman College of Nursing, Belmont University, Nashville, TN 37212, USA; david.phillippi@belmont.edu (D.P.); laura.gray@belmont.edu (L.G.); 4 School of Nursing, University of Alabama at Birmingham, Birmingham, AL 35233, USA; mahmadi@uab.edu (M.A.); abarmstrong@uab.edu (A.A.); elizcole@uab.edu (E.C.); schauf@uab.edu (M.R.); 5 Seattle Children’s Hospital, University of Washington, Seattle, WA 98105, USA; phillips@seattlechildrens.org; 6 Penn State Health Golisano Children’s Hospital, Pennsylvania State University, Hershey, PA 17033, USA; hsoistmann@pennstatehealth.psu.edu

**Keywords:** pediatric sleep health, family interaction, bedtime routine, outdoor play, ACEs

## Abstract

**Highlights:**

**What are the main findings?**

- The study’s large diverse sample is representative of children in the U.S. ages 3–5 years.
- Family activities (meals, reading, singing/telling stories) affect the relationship between adverse childhood experiences and sleep health outcomes in children ages 3–5 years.

**What are the implications of the main findings?**

- The effects of early adverse childhood experiences on pediatric sleep health may not be fully evident in the preschool years.
- Interactive family activities can have a positive effect on early childhood sleep health, even in the setting of adverse childhood experiences.

**Abstract:**

Background/Objectives: Sleep health is a multi-faceted biopsychosocial concept that affects well-being across the lifespan. Insufficient sleep occurs in large numbers of children and there is emerging evidence for the influence of socioecological factors and adverse childhood experiences (ACEs). The purpose of this study was to determine the influence of adverse childhood experiences on sleep health in preschool age children as mediated by family interaction habits and outdoor play. Methods: Our secondary analysis of the 2023 National Survey of Children’s Health used the weighted dataset filtered for children ages 3–5 years. Results: Sixty-six percent of parents reported adequate child sleep; nearly 89% of parents reported consistent bedtimes. Seventy-one percent reported no ACEs. Most children had one or more hours of outdoor play each day. Daily family interaction (reading, singing, stories, meals) occurred in approximately half of families. Structural equation modeling results indicated that the negative effects of ACEs on consistent bedtime were mediated by reading aloud and family meals, while adequate sleep duration was mediated by reading aloud and singing/storytelling. Conclusions: These findings represent a promising picture of young children and families, and insight into a window of time during child development when family interaction can positively affect sleep health in spite of adverse childhood experiences.

## 1. Introduction

Sleep health is a multi-faceted biopsychosocial concept encompassing dimensions (Behavior, Satisfaction, Alertness, Timing, Efficiency, Duration) that directly affect well-being across the lifespan [1,2,3,4,5]. Dimensions of sleep health include consistency of sleep habits that are formed early in life [6]. Guidelines for sufficient, regular sleep in early childhood help to guide parents and healthcare providers [7]; however, there are factors in a child’s life that may make healthy sleep difficult to attain, such as daily family routines and adverse childhood experiences (ACEs).

Insufficient sleep occurs in large numbers of children, from infancy through adolescence, and there is emerging evidence for socioecological disparities and the influence of adverse childhood experiences [8,9,10]. Evidence suggests that up to one-third of the pediatric population, ages 6–17 years, gets insufficient sleep, especially if they have experienced adverse childhood experiences (ACEs). This is particularly apparent if they have experienced multiple ACEs [9,10].

ACEs have been defined as events in a child’s life that are potentially traumatizing, such as witnessing violence or growing up with significant household dysfunction or instability [11,12]. While there is a body of evidence for the association of ACEs and sleep problems in adolescence and adulthood, little is known about the effect of ACEs on sleep in the younger child population [13,14,15]. Further, there are gaps in the literature regarding socioecological factors affecting children experiencing ACEs and sleep health problems [6,9,16].

Within the domain of child health, the concept of sleep health is reflected in the social-ecological framework of Billings et al. [1] in which sleep health is affected by a multidimensional realm of factors at the individual, family, and neighborhood level. In this framework, health and well-being are interrelated with sleep health. Individual factors include psychological and biological indicators; family factors include family interactions, family stress, and socioeconomic indicators [1,17].

There are critical points during child development that are especially important with regard to sleep health [18,19]. One of these points is the preschool years, when sleep onset and maintenance difficulties are more common [20]. There is limited sleep health research focused on this age group, particularly the investigation of specific individual, family, and socioecological factors such as ACEs, outdoor play and family activities that may be related to sufficient and consistent sleep habits [21].

Although increased physical activity has been shown to improve overall sleep quality in adolescents and adults, information specific to the effect on sleep in young children who have experienced ACEs is minimal [22]. Similarly, child and family factors influencing sleep quality and duration have been discovered in longitudinal studies across middle childhood and adolescence; however, there is a paucity of mediation analyses of these variables in early childhood [23]. Protective family factors (helpful child and family habits) conducive for sleep quality include warm relationships, support, structure with bedtimes and monitoring, while conflict, chaos, and family pressures may negatively impact sleep quality and duration [23].

**Figure 1:**
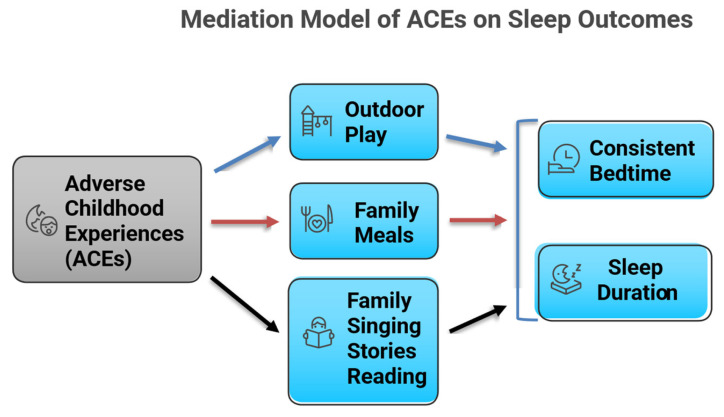
Conceptual model.

The purpose of this secondary data analysis of the 2023 National Survey of Children’s Health (NCHS) survey was to determine the influence of ACEs on sleep health in preschool age children as mediated by family interaction (family meals, singing, storytelling, and reading together) and child outdoor play. Using the socioecological and ACEs frameworks as a guide [1,11], we developed a conceptual model to represent our hypotheses about the mediating effect of variables on the relationship between ACEs and sleep health (see Figure 1). Our study focused on two specific sleep dimensions, consistent bedtime and sleep duration, with the goal of informing multidisciplinary pediatric healthcare providers and advocates. Our specific aims were as follows:

1. Describe ACEs, family interaction (family meals, singing/storytelling, reading together) and child outdoor play in families with children aged 3–5 years.
2. Characterize the relationship between ACEs and consistent bedtime and sleep duration in children aged 3–5 years.
3. Determine the mediating effect of the identified family interaction variables and child outdoor play on the relationship between ACEs and sleep health outcomes.

## 2. Materials and Methods

A secondary analysis was performed using data from the 2023 NSCH survey as part of the Child and Adolescent Health Measurement Initiative (CAHMI). Permission to download and use the data file for this study was obtained from the Data Resource Center for the Child and Adolescent Health Measurement Initiative (CAHMI) [24]. This dataset is publicly available through CAHMI. The NSCH is a survey that includes data related to the health and well-being of children in the United States, including family interactions, parental health, school and after-school experiences, and neighborhood characteristics.

The 2023 survey was administered nationally June 2023 until January 2024 with a total of 55,162 questionnaires completed by parents/caregivers of children and youth, ages 0–17 years of age [24]. The sampling design used United States Census Bureau data to achieve weighting that reflected characteristics of each state’s population of non-institutionalized children aged 0–17. Only one child per household was randomly selected to be the subject of the detailed questionnaire [25].

Our two outcome variables, adequate sleep duration and consistent bedtime, are dimensions related to sleep health. Although there are multiple dimensions of sleep health that exist, these were the only two sleep variables measured in the 2023 NSCH other than infant sleep position [26]. In the survey, adequate sleep duration was operationalized as the amount of sleep the child gets on an average day, based on the 10 to 13 recommended hours of sleep for children aged 3–5, including naps [7]; it was coded as 1 = yes if the response indicated 10, 11, or 12+ h and 0 = no if sleep amount was less than the recommended parameters. Consistent bedtime was operationalized in the survey as “how often does this child go to bed at about the same time on weeknights,” originally measured on an ordinal scale from 1, “always”, to 4, “rarely or never”. These responses were transformed into a dichotomous outcome, recoded to 1 = yes if the response indicated “always” or “usually” and 0 = no if the response indicated “sometimes” or “rarely or never.”

The number of ACEs served as the single independent variable of interest. The measurement of ACEs that was used pertained to a composite of household and community-based items such as: hard to cover basics on family’s income; parent or guardian divorced or separated; parent or guardian died; parent or guardian served time in jail; saw or heard parents or adults slap, hit, kick punch one another in the home; was a victim of violence or witnessed violence in their neighborhood; lived with anyone who was mentally ill, suicidal, or severely depressed; lived with anyone who had a problem with alcohol or drugs; treated or judged unfairly due to race/ethnicity; and treated unfairly because of a health condition or disability. The number of ACEs in the composite variable ranged from 0, “no adverse childhood experiences”, to 10 [26].

Five pertinent mediating variables that relate to ACEs and sleep were examined: play outside on weekdays, play outside on weekends, how often the family reads to the child, how often the family sings or tells stories to the child, and frequency of family meals together. These variables were operationalized for the 3–5-year-old age group per NSCH definitions [26] (pp. 30–31, 250–252, 257, 275, 276), with higher values indicating greater frequency as follows:

1. Outdoor play on weekdays—“On most weekdays, how much time does this child spend playing outdoors?” Response options specified one hour or less per day, two hours per day, three hours per day, or four or more hours per day.
2. Outdoor play on weekends—“On an average weekend, how much time does this child spend playing outdoors?” Response options specified one hour or less per day, two hours per day, three hours per day, or four or more hours per day.
3. Family reading—“During the past week, how many days did you or other family members read to this child?” Response options specified no days, one to three days, four to six days, or every day.
4. Stories and songs—“During the past week, how many days did you or other family members tell stories or sing songs to this child?” Response options specified no days, one to three days, four to six days, or every day.
5. Family meals—“During the past week, on how many days did all the family members who live in the household eat a meal together?” Response options specified no days, one to three days, four to six days, or every day.

Additionally, five covariates were included in our two statistical models to control for differences due to demographic characteristics in the study sample. These were chosen based on the demographic variables utilized by the NSCH for weighting the observations: sex, race/ethnicity, income relative to state poverty level, parents’ highest level of education, and number of children in the household [25]. The variable for sex was dummy coded with male as the baseline (0 = male), as was race/ethnicity with white as the baseline (0 = white). Due to low frequencies within two race categories for American Indian or Alaska Native and Native Hawaiian and Other Pacific Islander, these two groups were combined into a single category. The three other covariates were treated as continuous due to their ordinal measurement levels.

Two single parallel structural equation models (SEMs) were used to estimate the mediating effects that outdoor play, family reading, family singing/storytelling, and family meals have on the relationship between ACEs and the two sleep outcomes of interest. Due to the survey’s complex design that included stratification, weights, and primary sampling units (PSUs), R’s lavaan and lavaan.survey packages were used to specify and adjust for the survey design. The models were run using weighted least squares mean and variance adjusted estimators, as this logistic/probit framework is most appropriate for samples ≥ 200 with binary or ordinal outcome variables within SEM [26,27,28].

RStudio, Version 2026.01.2+418 was used to analyze the data, including the lavaan, lavaaan.survey, and survey packages. The NSCH weighted dataset was filtered to include only children between the ages of 3–5 years, after which the dataset’s weights were adjusted using SPSS (version 31.0.0.0) weighting command “adjust weights by age” to accommodate the smaller sample size of 12,055 cases, preventing any sample size inflation.

## 3. Results

The final sample size consisted of 12,055 children who had an average age of 4.00 (*SD* = 0.81). The sample had slightly more males (51.2%, *n* = 6176) than females (48.8%, *n* = 5879), with a race/ethnicity comprising mostly white (48.6%, *n* = 5857) or Hispanic (26.0%, *n* = 3135). Most participants had an income level that was at or above poverty level (82.0%, *n* = 9890), most households had 1 to 2 children (62.2%, *n* = 9508), and the majority of parents had more than a high school education (75.0%, *n* = 9036). A more detailed summary of these demographics, both weighted and unweighted, can be found in Table 1.

Regarding the sleep outcomes of interest, most parents reported a consistent bedtime (88.9%, *n* = 10,504), and most reported their child slept the recommended number of hours (labeled adequate sleep duration) for their age (66.2%, *n* = 7836). Most participants reported no ACEs (70.8%, *n* = 8308), while 18.8% reported 1 ACE (*n* = 2206), 9.2% reported 2–4 ACEs (*n* = 1083), and 1.5% reported five or more ACEs (*n* = 135). Almost half of parents reported that they sang or told stories to their child every day (45.4%, *n* = 5316), 42.1% read aloud to their children every day (*n* = 4927), and 59.6% ate at least one meal together every day (*n* = 6967). On weekends, only a small percentage played outdoors less than 1 h per day (6.3%, *n* = 736), while most played for 1–2 h (43.5%, *n* = 5058) or at least 3 h (50.2%, *n* = 5836). Similarly, on weekdays only a small percentage played outdoors less than 1 h per day (8.8%, *n* = 1026), while most played for 1–2 h (61.4%, *n* = 7142) or at least 3 h (29.7%, *n* = 3456). All statistics reported throughout this study are based on weighted results calculated over valid (non-missing) responses. A more detailed summary of the dependent, independent, and mediator variables can be found in Table 2.

### Mediating Sleep Outcomes

**Figure 2:**
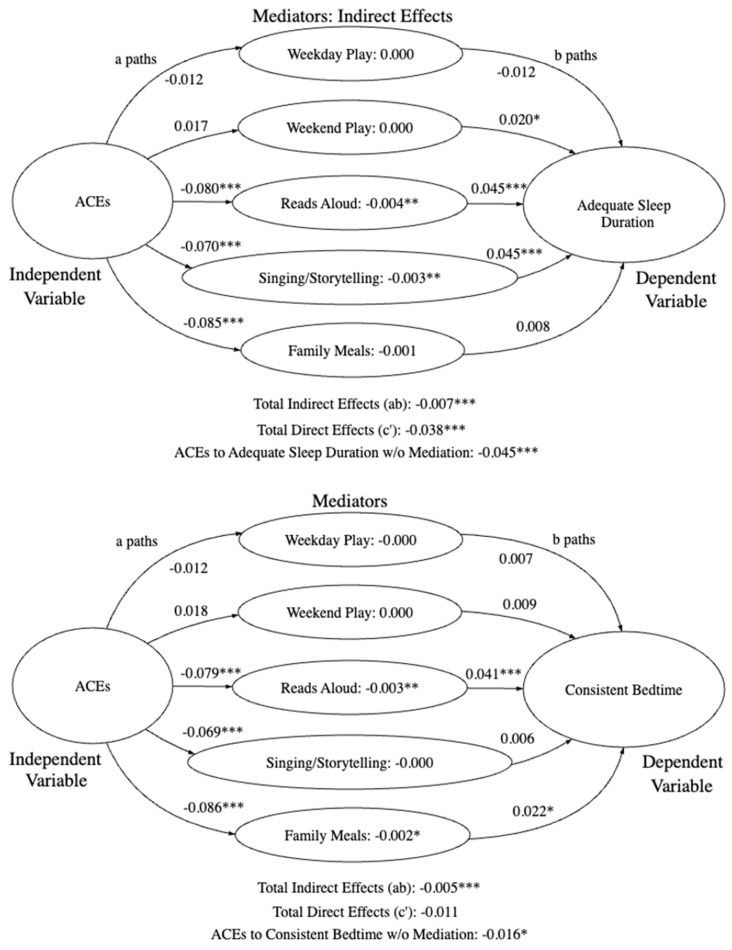
Pathways for both SEMs with significance denoted. * *p* < 0.05; ** *p* < 0.01; *** *p* < 0.001.

The two mediation relationships were estimated through parallel structural equation modeling (SEM), adjusted for the complex survey design. Both models examined whether the five mediator variables (weekday play, weekend play, reading to the child, singing/telling stories, and eating meals together) mediated the relationship between the independent variable (ACEs) and the dependent variable (consistent bedtime or adequate sleep duration), while controlling for the covariates (sex, race/ethnicity, income relative to poverty, education, and number of children in the household). Note that while the term effect size is often used to describe numerical relationships or associations between variables in SEM, this does not imply a causal relationship in our study. A summary of the two models and their pathways can be found in Table 3 and Figure 2, respectively.

When modeling adequate sleep duration, the direct paths from the independent variable to the mediators (the “a” paths) revealed that a higher number of ACEs were associated with a decrease in three family interactions, while controlling for covariates. Specifically, more ACEs were associated with fewer family meals together (B = − 0.085, SE = 0.017, z = −5.101, *p* < 0.001), less frequent reading to the child (B = −0.080, SE = 0.018, z = −4.518, *p* < 0.001), and less frequent singing or storytelling (B = −0.070, SE = 0.018, z = −3.815, *p* < 0.001). The effect of ACEs on child outdoor play was insignificant.

The direct paths from the mediators to the dependent variable (the “b” paths) revealed that three mediating family interactions significantly increased the likelihood of a child getting an adequate amount of sleep, while controlling for covariates. Specifically, singing/storytelling (B = 0.045, SE = 0.011, z = 4.196, *p* < 0.001), reading to the child (B = 0.045, SE = 0.011, z = 3.956, *p* < 0.001), and outdoor weekend play (B = 0.020, SE = 0.009, z = 2.185, *p* = 0.029) were associated with a higher likelihood of adequate sleep duration.

Two indirect paths from the independent variable to the dependent variable through the mediator variables (the “ab” paths) had a significant total effect, while controlling for covariates (B = −0.007, SE = 0.002, z = −3.552, *p* < 0.001). Specifically, the mediators for reading to the child (B = −0.004, SE = 0.000, z = −2.806, *p* = 0.005) and singing/storytelling (B = −0.003, SE = 0.001, z = −2.787, *p* = 0.005) both significantly reduced the negative effects that ACEs had on the likelihood of adequate sleep duration.

Overall, the significant relationship between ACEs and receiving an adequate amount of sleep (B = −0.045, SE = 0.009, z = −5.036, *p* < 0.001) was partially mediated by reading aloud and singing/storytelling with the child, while controlling for covariates (B = −0.038, SE = 0.009, z = −4.296, *p* < 0.001). According to standardized estimates, 18.5% of the total direct effects (β = −0.081) were attributed to the mediation’s indirect effects (β = −0.015).

Results for consistent bedtime were somewhat similar, but with full mediation. The direct paths from the independent variable to the mediators (the “a” paths) revealed that more ACEs were associated with a decrease in three family interactions, while controlling for covariates. Specifically, more ACEs were associated with fewer family meals together (B = −0.086, SE = 0.017, z = −5.148, *p* < 0.001), less frequent reading to the child (B = −0.079, SE = 0.018, z = −4.484, *p* < 0.001), and less frequent singing or storytelling (B = −0.069, SE = 0.018, z = −3.737, *p* < 0.001). The effect of ACEs on child outdoor play was insignificant.

The direct paths from the mediators to the dependent variable (the “b” paths) revealed that two of the mediating family interactions significantly increased the likelihood of a child having a consistent bedtime, while controlling for covariates. Specifically, reading to the child (B = 0.041, SE = 0.007, z = 5.645, *p* < 0.001) and eating family meals together (B = 0.022, SE = 0.009, z = 2.434, *p* = 0.015) were associated with a higher likelihood of a consistent bedtime.

Two indirect paths from the independent variable to the dependent variable through the mediator variables (the “ab” paths) had a significant total effect, while controlling for covariates (B = −0.005, SE = 0.001, z = −3.865, *p* < 0.001). Specifically, reading to the child (B = −0.003, SE = 0.001, z = −3.455, *p* = 0.001) and eating family meals together (B = −0.002, SE = 0.001, z = −2.160, *p* = 0.031) both significantly reduced these negative effects that ACEs had on the likelihood of a consistent bedtime.

Overall, the significant relationship between ACEs and a consistent bedtime (B = −0.016, SE = 0.007, z = −2.191, *p* = 0.028) was fully mediated by reading aloud to the child and family meals together, while controlling for covariates (B = −0.011, SE = 0.007, z = −1.452, *p* = 0.146). The change from ACEs having a significant relationship with consistent bedtime without mediation to non-significance with mediation indicates that the relationship is fully mediated. According to standardized estimates, 50.0% of the total direct effects (β = −0.034) were attributed to the mediation’s indirect effects (β = −0.017).

## 4. Discussion

This study examined sleep health outcomes in a large sample of children (N = 12,055) at ages 3–5 years, an early childhood developmental phase known for building sleep habits. While there are a multitude of biopsychosocial factors observed in the 2023 National Child Health Survey, we chose to examine specific and potentially modifiable socioecological factors (family interaction activities and child outdoor play) in the context of ACEs and sleep health outcomes. Most of the parents in the sample reported adequate amounts of child sleep, consistent bedtimes, and the absence of ACEs. Analysis of our variables of interest revealed significant negative effects of ACEs on adequate sleep duration and consistent bedtime. We also discovered evidence for the beneficial effects of several child and family routines on specific sleep outcomes, even in the setting of ACEs in families with young children. It is relevant to note that our statistical models controlled for pertinent socioecological covariates in the population that may affect the relationships. The implications of these results are informative for families and early childhood professionals when discussing sleep health interventions; however, they should be interpreted with caution in light of small effect sizes. Our findings can be synthesized in the context of the extant literature.

### 4.1. ACEs and Sleep

Our analysis found a statistically significant association between the number of ACEs and adequate sleep duration and consistent bedtime in preschool-age children. The majority (70.8%) of children in our analysis had no ACEs reported; however, our model demonstrated that the more ACEs present, the greater the likelihood of inadequate sleep duration and inconsistent bedtime. Similarly, Song and colleagues [10], in a secondary analysis of the 2020–2021 NSCH data for 6–17-year-old children, found that for every increase in ACES there was an increased likelihood of suboptimal sleep duration. Children aged 6–12 years tended to sleep less than the recommended hours of sleep while children 13–17 tended to sleep more than the recommended hours of sleep.

These findings can also be interpreted in light of the contrasting results from the study by Greeson and colleagues [26,27,28] utilizing data from the National Child Traumatic Stress Network database in which there was a dose–response effect of ACEs on child behavior problems, but not sleep problems. The children in Greeson’s study ranged from toddler through adolescent age, and sleep problems were described using the Child Behavior Checklist, a tool that reflects multiple dimensions of sleep health per parent report [29]. Our large study sample described a much narrower age range and suggests a possible dose–response effect of ACEs on two dimensions of sleep (parent-reported timing and duration); however, with small effect sizes the association may yet to be actualized at the preschool age. There is evidence in other studies that increasing effects may emerge in later childhood or adolescence [30,31,32].

Adequate sleep duration in the NSCH surveys is assessed by parent report of sufficient amount of sleep according to age per the guidelines from the American Academy of Sleep Medicine [7]. These guidelines recommend 12–16 h of sleep per 24 h for 4–12-month-olds; 11–14 h for 1–2-year-olds; 10–13 h for 3–5-year-olds; and 9–12 h for 6–12-year-olds. Knowledge of age-appropriate sleep duration is integral to a family’s ability to promote child wellbeing, flourishing, cardiovascular health, metabolism, immune status, mental health and performance [4,7].

### 4.2. Outdoor Play, ACEs, and Sleep

The NSCH data provides insight into real-life frequency of outdoor play, and more than 90% of the sample played at least one hour per day outdoors on both weekdays and weekends. The effect that ACEs had on outside play on weekdays and weekends in this study was insignificant, as was the mediating effect of play on the relationship between ACEs and sleep. Interestingly, outdoor weekend play was associated with a higher likelihood of adequate sleep duration. Few studies have examined play outside on weekdays or weekend days or physical activity as a mediator of the relationships of ACES and hours of sleep or consistent bedtime, particularly in 3–5-year-olds. Lewis-de los Angeles [33] examined the association of ACES and sleep and exercise in 10–11-year-olds and found an association with sleep but not with exercise. The investigator did not examine the mediating effect of exercise on the relationship between ACES and sleep.

Song and colleagues [10], in a secondary data analysis of data from the 2020–2021 NSCH for 6–17-year-old children, found children who were physically active had lower prevalence of suboptimal sleep duration. This was a confirmation of findings of a meta-analysis by Lang et al. [22] who noted that, in mid and older adolescence, those with higher subjective and objective physical activity levels had better sleep. It may be that the associations of ACES, physical activity, and sleep are not yet apparent in 3–5-year-olds. Further, without evidence from other studies, it is difficult to determine if outdoor play mediates the relationship between ACES and sleep in other age groups.

There is evidence in younger children that adequate sleep, combined with light to moderate physical activity, improves cognitive function, specifically working memory; moreover, younger children require higher amounts of physical activity for this effect compared to older children and adolescents [34]. However, the effect of activity on sleep health may vary according to socioeconomic status [34,35,36], thus child health advocates should incorporate cultural sensitivity when partnering with parents about their child’s physical activity and sleep health goals. With awareness of the possible confounding effects of socioeconomic factors, our analysis controlled for these covariates.

### 4.3. Family Meals, ACEs and Sleep

Our study found higher ACE counts were associated with fewer family meals together. In addition, family meals had a significant mediating effect on the relationship between ACEs and consistent bedtime but not adequate sleep duration. Establishing consistent and routine meals together as a family has been shown to increase sleep quality and sleep health [37]. There is no standard measure of family meals, but it is typically measured by the number of days in a week that a family eats together and may differentiate which meals are eaten together [37,38,39]. In the systematic review of Snugs and Harvey [39] there was evidence for the positive association of family mealtimes and child/adolescent psychological wellbeing. While this study did not include ACEs specifically or sleep, this relationship could be a clue to a positive effect of family meals together on sleep in the setting of ACEs. The association of family meals impacting sleep has not been well studied in younger children; however, there are data in older age groups indicating family meals may improve sleep duration. Godos and colleagues [40] examined the impact of diet, eating habits and lifestyle on sleep duration in children 6–17 years old from Mediterranean countries, and found that family meals together were associated with adequate sleep duration.

### 4.4. Family Reading, Singing/Storytelling, ACEs, and Sleep

In addition to family meals together, our study revealed that higher ACE counts were associated with fewer family interactions that involved reading, singing, and storytelling. Reading together had a significant effect on the relationship between ACEs and both of our sleep health outcomes. Similarly, singing and storytelling improved the likelihood of children affected by ACEs getting adequate amounts of sleep. It is important to note that the NSCH survey did not specify the timing of the family interactions during the day.

Although there is a lack of evidence in the literature for similar findings involving ACEs in preschool children, there are recent studies linking family routines to overall child mental, psychological, and physical wellbeing, including the positive effect of bedtime routines on sleep [41,42]. These findings also align with evidence from prior sleep research in preschool children, including longitudinal studies, that show family singing and reading together can serve as important protective factors for preschool children’s sleep health and wellbeing. Shared bedtime activities such as reading stories and engaging in calm verbal interactions have been associated with longer nighttime sleep and improved sleep outcomes in preschool children [43,44]. A consistent bedtime routine is linked to multiple beneficial sleep outcomes, including longer sleep duration, improved sleep regulation, and better overall sleep patterns among children aged three to five years [44,45,46]. Calming parent–child interactions at bedtime may help children relax and prepare physiologically and emotionally for sleep, and there is evidence that the earlier in preschool years these bedtime routines begin, the better the child’s sleep health as they grow older [45]. This calming effect may be involved in the influence of family interaction on the relationship of ACEs and sleep in young children.

### 4.5. Limitations

The data analyzed for this study were captured near the end of the COVID-19 pandemic in 2023; therefore, the effect of this event on several of the predictors and outcomes of this study should be acknowledged. For example, disruption of families’ home environments and routines may constitute an adverse child experience. In addition, the ACEs composite measure utilized for this study may not capture the daily stressors of the child’s current family environment. Another example would be the stressful process of family conflict prior to parental separation that may not be captured in the survey’s ACEs measurement item.

Another limitation is related to the measure of physical activity. The NSCH quantifies activity in the 3–5-year-old age group based on parent reports of outdoor play hours. This method of measurement is not sensitive to the specific activity level of the child. Likewise, an additional measurement limitation in the NSCH is the method of parent report of sleep, and the inclusion of only two dimensions of sleep on the survey, as sleep is a multidimensional construct.

## 5. Conclusions

The results from our study reflect a large representative sample of children in the United States ages 3–5 years whose parents participated in the National Survey of Children’s Health in 2023. The findings are generalizable due to the large sample size, are representative of a diverse population, and have implications for children in the preschool years and their families. While this study is cross-sectional in nature and cannot establish causal relationships, our analysis was rigorously adjusted for potential confounding variables including race/ethnicity, sex, household poverty, parent education, and household size. This adjustment strengthened the insight about the positive effects that family interactions have on child sleep health outcomes. Activities of meaningful family interaction (meals, singing, stories, reading together) had small but significant effects on the relationship between ACEs and two dimensions of sleep health, evidence that is relevant for child sleep health promotion at this stage of child development.

Future research may yield more clinically significant findings by including improved measures of physical activity and adequate sleep duration in children. Ideally these measures would include objective physiologic activity monitoring or detailed observation. Future longitudinal research is especially indicated in the focus on family activities and healthy sleep throughout childhood, and will inform the early establishment of routines that are essential to child well-being and sleep health. Family-centered study designs should address the many influences in a child’s life beginning prenatally, such as parenting style, transgenerational effects, socioecological, socioeconomic, and geographic factors.

It is promising that the adverse experiences of the preschool children in this study may not yet have had a major effect on sleep health, suggesting a window of time that is prime for anticipatory guidance with families. Optimizing child and family routines in this particular developmental stage (early childhood) may help decrease the effects of ACEs on sleep health. Based on the results of our study, together with the body of evidence in the literature, it is important that families know about the potential positive effects of consistent child outdoor play, family mealtimes and nurturing family interaction on child sleep health. Beginning these activities as early as possible in childhood can create a foundation for sleep health which in turn promotes wellbeing. Further exploration of modifiable socioecological factors at the family level during the preschool years in the setting of adverse childhood experiences can provide insight into the resiliency of young children in nurturing family environments.

## Figures and Tables

**Table 1:** Summary of demographic variables.

		Unweighted		Weighted	
Demographic Variable		*n*	*%*	*n*	*%*
Sex					
	Male	6085	50.5	6176	51.2
	Female	5970	49.5	5879	48.8
Race/Ethnicity					
	White	7923	65.7	5857	48.6
	Hispanic	1700	14.1	3135	26.0
	Black	655	5.4	1387	11.5
	Asian	675	5.6	559	4.6
	Multi-Race	1014	8.4	1034	8.6
	American Indian/Alaska Native	69	0.6	68	0.6
	Native Hawaiian/Pacific Islander	19	0.2	15	0.1
Income Relative to State Poverty Level					
	0–99%	1355	11.2	2166	18.0
	100–199%	1825	15.1	2281	18.9
	200–399%	3645	30.2	3578	29.7
	400%+	5230	43.4	4031	33.4
Highest Level of Education					
	Less than High School	203	1.7	814	6.8
	High School/Vocational/Trade School	1335	11.1	2204	18.3
	More than High School	10,517	87.2	9036	75.0
Number of Children in Household					
	1	3952	32.8	2434	20.2
	2	5556	46.1	5066	42.0
	3	1916	15.9	3007	24.9
	4+	631	5.2	1548	12.8

*Note.* N = 12,055.

**Table 2:** Summary of dependent, independent, and mediator variables.

		Unweighted		Weighted	
Dependent Variables		*n*	*%*	*n*	*%*
Consistent Bedtime		N = 11,912		N = 11,811	
	Yes	10,859	91.2	10,504	88.9
	No	1053	8.8	1306	11.1
Adequate Sleep Duration		N = 11,938		N = 11,833	
	Yes	8433	70.6	7836	66.2
	No	3505	29.4	3997	33.8
**Independent Variable**					
Adverse Childhood Experiences—ACEs		N = 11,830		N = 11,733	
	0	8875	75.0	8308	70.8
	1	1890	16.0	2206	18.8
	2–4	952	8.0	1083	9.2
	5+	113	1.0	135	1.5
**Mediator Variables**					
Outdoor Play on Weekends		N = 11,802		N = 11,629	
	<1 h	516	4.4	736	6.3
	1 h	1628	13.8	1881	16.2
	2 h	3154	26.7	3177	27.3
	3 h	3116	26.4	2821	24.3
	4+ h	3388	28.7	3015	25.9
Outdoor Play on Weekdays		N = 11,796		N = 11,623	
	<1 h	744	6.3	1026	8.8
	1 h	2912	24.7	3046	26.2
	2 h	4230	35.9	4096	35.2
	3 h	2409	20.4	2135	18.4
	4+ h	1501	12.7	1321	11.4
Read Aloud to Child		N = 11,826		N = 11,709	
	0 Days	440	3.7	628	5.4
	1–3 Days	3264	27.6	4147	35.4
	4–6 Days	2193	18.5	2008	17.1
	Every Day	5929	50.1	4927	42.1
Sung or Told Stories to Child		N = 11,815		N = 11,708	
	0 Days	399	3.4	507	4.3
	1–3 Days	3178	26.9	3610	30.8
	4–6 Days	2327	19.7	2276	19.4
	Every Day	5911	50.0	5316	45.4
Ate Meal Together with Child		N = 11,812		N = 11,696	
	0 Days	210	1.8	252	2.2
	1–3 Days	1732	14.7	1825	15.6
	4–6 Days	3033	25.7	2653	22.7
	Every Day	6837	57.9	6967	59.6

*Note.* Weighted and unweighted sample sizes indicated per variable.

**Table 3:** Summary of the results from the SEM for adequate sleep duration and consistent bedtime.

Pathways and Relationships		*B*	β	*SE*	*z*	*p*
Adequate Sleep Duration						
	Total Direct Effects (c’)	−0.038	−0.081	0.009	−4.296	<0.001 ***
	Total Indirect Mediation Effects (ab)	−0.007	−0.015	0.002	−3.552	<0.001 ***
	Indirect Mediation Effects: Weekday Play (ab1)	0.000	0.000	0.000	0.543	0.587
	Indirect Mediation Effects: Weekend Play (ab2)	0.000	0.001	0.000	0.696	0.486
	Indirect Mediation Effects: Reading Aloud(ab3)	−0.004	−0.008	0.000	−2.806	0.005 **
	Indirect Mediation Effects: Singing/Storytelling (ab4)	−0.003	−0.007	0.001	−2.787	0.005 **
	Indirect Mediation Effects: Family Meals (ab5)	−0.001	−0.001	0.001	−0.740	0.459
	ACEs to Weekday Play (a1)	−0.012	−0.011	0.021	−0.595	0.552
	ACEs to Weekend Play (a2)	0.017	0.014	0.023	0.717	0.473
	ACEs to Reading Aloud (a3)	−0.080	−0.080	0.018	−4.518	<0.001 ***
	ACEs to Singing/Storytelling (a4)	−0.070	−0.072	0.018	−3.815	<0.001 ***
	ACEs to Family Meals (a5)	−0.085	−0.102	0.017	−5.101	<0.001 ***
	Weekday Play to Adequate Sleep (b1)	−0.012	−0.029	0.010	−1.285	0.199
	Weekend Play to Adequate Sleep (b2)	0.020	0.050	0.009	2.185	0.029 *
	Reading Aloud to Adequate Sleep (b3)	0.045	0.095	0.011	3.956	<0.001 ***
	Singing/Storytelling to Adequate Sleep (b4)	0.045	0.093	0.011	4.196	<0.001 ***
	Family Meals to Adequate Sleep (b5)	0.008	0.014	0.011	0.748	0.454
	ACEs to Adequate Sleep without Mediation (c)	−0.045	−0.095	0.009	−5.036	<0.001 ***
Consistent Bedtime						
	Total Direct Effects (c’)	−0.011	−0.034	0.007	−1.452	0.146
	Total Indirect Mediation Effects (ab)	−0.005	−0.017	0.001	−3.865	<0.001 ***
	Indirect Mediation Effects: Weekday Play (ab1)	−0.000	−0.000	0.000	−0.466	0.641
	Indirect Mediation Effects: Weekend Play (ab2)	0.000	0.000	0.000	0.618	0.536
	Indirect Mediation Effects: Reading Aloud (ab3)	−0.003	−0.010	0.001	−3.455	0.001 **
	Indirect Mediation Effects: Singing/Storytelling (ab4)	−0.000	−0.001	0.001	−0.691	0.490
	Indirect Mediation Effects: Family Meals (ab5)	−0.002	−0.006	0.001	−2.160	0.031 *
	ACEs to Weekday Play (a1)	−0.012	−0.010	0.021	−0.570	0.569
	ACEs to Weekend Play (a2)	0.018	0.014	0.024	0.755	0.450
	ACEs to Reading Aloud (a3)	−0.079	−0.080	0.018	−4.484	<0.001 ***
	ACEs to Singing/Storytelling (a4)	−0.069	−0.071	0.018	−3.737	<0.001 ***
	ACEs to Family Meals (a5)	−0.086	−0.103	0.017	−5.148	<0.001 ***
	Weekday Play to Consistent Bedtime (b1)	0.007	0.023	0.008	0.793	0.427
	Weekend Play to Consistent Bedtime (b2)	0.009	0.033	0.007	1.159	0.247
	Reading Aloud to Consistent Bedtime (b3)	0.041	0.131	0.007	5.645	<0.001 ***
	Singing/Storytelling to Consistent Bedtime (b4)	0.006	0.017	0.008	0.692	0.489
	Family Meals to Consistent Bedtime (b5)	0.022	0.057	0.009	2.434	0.015 *
	ACEs to Consistent Bedtime without Mediation (c)	−0.016	−0.051	0.007	−2.191	0.028 *

*Note.* N = 12,055. * *p* < 0.05; ** *p* < 0.01; *** *p* < 0.001.

## Data Availability

Permission to download and use the data file for this study was obtained from the Data Resource Center for the Child and Adolescent Health Measurement Initiative (CAHMI) [24]. This dataset is publicly available through CAHMI.

## Abbreviations

The following abbreviations are used in this manuscript:

ACE	Adverse Childhood Experience
NSCH	National Survey of Children’s Health
